# Factors influencing the reliability of intraoperative testing in deep brain stimulation for Parkinson’s disease

**DOI:** 10.1007/s00701-023-05624-4

**Published:** 2023-06-02

**Authors:** Tobias Mederer, Daniel Deuter, Elisabeth Bründl, Patricia Forras, Nils Ole Schmidt, Zacharias Kohl, Jürgen Schlaier

**Affiliations:** 1 Department of Neurosurgery, University Hospital Regensburg, Franz-Josef-Strauß Allee 11, 93053 Regensburg, Germany; 2 Regensburg Regional Hospital for Forensic Health Psychiatry and Neurology, Universitätsstraße 84, 93053 Regensburg, Germany

**Keywords:** Deep brain stimulation; Parkinson’s disease, Subthalamic nucleus, Intraoperative clinical testing

## Abstract

**Background:**

Several meta-analyses comparing the outcome of awake versus asleep deep brain stimulation procedures could not reveal significant differences concerning the postoperative improvement of motor symptoms. Only rarely information on the procedural details is provided for awake operations and how often somnolence and disorientation occurred, which might hamper the reliability of intraoperative clinical testing. The aim of our study was to investigate possible influencing factors on the occurrence of somnolence and disorientation in awake DBS procedures.

**Methods:**

We retrospectively analyzed 122 patients with Parkinson's disease having received implantation of a DBS system at our centre. Correlation analyses were performed for the duration of disease prior to surgery, number of microelectrode trajectories, AC-PC-coordinates of the planned target, UPDRS-scores, intraoperative application of sedative drugs, duration of the surgical procedure, perioperative application of apomorphine, and the preoperative L-DOPA equivalence dosage with the occurrence of intraoperative somnolence and disorientation.

**Results:**

Patients with intraoperative somnolence were significantly older (*p*=0.039). Increased duration of the DBS procedure (*p*=0.020), delayed start of the surgery (*p*=0.049), higher number of MER trajectories (*p*=0.041), and the patients’ % UPDRS improvement (*p*=0.046) also correlated with the incidence of intraoperative somnolence. We identified the main contributing factor to intraoperative somnolence as the use of sedative drugs applied during skin incision and burr hole trepanation (*p*=0.019). Perioperatively applied apomorphine could reduce the occurrence of somnolent phases during the operation (*p*=0.026).

**Conclusion:**

Several influencing factors were found to seemingly increase the risk of intraoperative somnolence and disorientation, while the use of sedative drugs seems to be the main contributing factor. We argue that awake DBS procedures should omit the use of sedatives for best clinical outcome. When reporting on awake DBS surgery these factors should be considered and adjusted for, to permit reliable interpretation and comparison of DBS study results.

**Supplementary Information:**

The online version contains supplementary material available at 10.1007/s00701-023-05624-4.

## Introduction

In the beginning of the era of deep brain stimulation (DBS) in Parkinson´s disease (PD) most procedures were performed with the patient awake for intra-operative clinical testing [26] [12] [1]. The purpose of clinical testing is to identify the optimal stimulation site, defined by the best reduction of major motor symptoms, while avoiding any side effects. However, the necessity of operations with the patient awake has been challenged early on [2] and an increasing number of implantations nowadays is performed under general anesthesia, guided by anatomical targeting alone or in combination with microelectrode (MER) recordings. Publications concerning the usefulness of intra-operative clinical testing show heterogeneous results [33][6][14][20][13] [8]. In fact, a prospective, randomized clinical trial [19] and several meta-analyses did not reveal significant differences concerning the improvement of motor symptoms when comparing the outcome of awake versus asleep DBS procedures [22][9][18, 27, 37]. Although, a wide range of anesthesiological approaches and perioperative managements have been described for “awake procedures” [28, 29, 36][24], they are rarely reported in detail when comparing awake versus asleep approaches. During awake procedures, intraoperative drowsiness, somnolence and disorientation might occur with an incidence of 1–33% [4, 11, 15, 25, 31]. This potentially hampers the reliability of the determination of the optimal stimulation site and diminishes the advantages of intraoperative clinical testing compared to operations under general anesthesia.

The aim of our retrospective study is to investigate which factors influence the occurrence of somnolence and disorientation during the awake operation. We provide information that will help to improve the reliability and usefulness of awake procedures in the future.

## Materials and methods

A total of 122 patients with PD who underwent bilateral subthalamic nucleus-targeted deep brain stimulation (STN-DBS) between 2002 and 2020 were analyzed in this retrospective, observational study. Patient age ranged from 42 to 75 years (mean: 61.8 years). The mean interval between the first diagnosis of PD and DBS surgery was 11.3 years (range: 2–25 years). 57 patients presented with equivalent type, 48 with hypokinetic-rigid type and 17 with tremor-dominant type of PD. Two patients with intracerebral hemorrhages, documented on postoperative CT scans, were not included in this study.

A multidisciplinary team of trained neurologists, neurosurgeons and psychiatrists evaluated the diagnosis of PD and eligibility to receive DBS based on several preoperative tests. Patients with severe brain atrophy, psychiatric disorders and other serious conditions were excluded. All data was acquired during our standard preoperative examinations for DBS surgery. No additional image acquisition or patient testing was conducted. The study was approved by the local ethics committee at the University Medical Center and conducted in accordance with the Declaration of Helsinki.

### L-DOPA withdrawal prior to the operation

From 2002 to 2008, all patients (*n*=41) were put on L-DOPA monotherapy several days prior to the operation. In the afternoon before surgery, all PD related medication was withdrawn until completion of the operation. From 2008 to 2020 (81 patients), PD medication were replaced by L-DOPA monotherapy 10 days prior to DBS surgery, but on admittance the oral L-DOPA medication was substituted by continuous subcutaneous apomorphine treatment. The apomorphine pump was stopped 1h prior to surgery and continued immediately after completion of the operation.

### Timing of the pre-operative MRI scan

The first 11 patients received their preoperative stereotactic MRI scan on the day of surgery under general anesthesia with a ceramic head-ring and fiducials attached. In all later patients (*n* =111) MRI was acquired one or two days prior to the operation under general anesthesia to reduce movement artefacts for optimal surgical planning. The DBS protocol included structural sagittal T1-weighted (1-mm slice thickness) and axial and sagittal T2-weighted (2 mm slice thickness) MRI sequences for trajectory planning. On the day of surgery, a CT scan was performed with a mounted stereotactic frame and fused to the MRI data including the planned trajectories. Target planning was based on MRI anatomy rather than atlas-coordinates in all patients, according to the publication of Bejjani [3]. Nevertheless, atlas coordinates for the central trajectory, related to the mid-commissural point, were determined and documented, as well as the width of the third ventricle. We sought to apply five parallel microelectrode trajectories for each side, but omitted one or more trajectories, if critical cerebral structures were threatened by placing of the electrodes (e.g., blood vessels, sulci, ventricles). For postoperative imaging, a CT scan with 1-mm slice thickness was performed to verify surgery results.

### Anesthesiologic regimen

Intraoperative microelectrode recording (MER) and clinical testing was performed in a patient-awake state in all cases. However, from 2002 to 2006 (21 patients), the procedure followed the asleep-awake-asleep-awake protocol with patients receiving sedation (remifentanil and propofol) for skin incision and burr hole trepanation. Sedative drugs were stopped prior to MER and clinical testing.

All subsequent patients were operated with psychological support by the anesthesiologist preferably without any sedative drugs according our awake-awake-awake-protocol [39].

### Intraoperative clinical testing

Clinical testing was performed with the macro tip of the microelectrodes (FHC, Bowdoin, ME) starting with the trajectory, that matched best to the anterior-superior-lateral (“sensorimotor”) part of the STN [24]. Depending on the clinical thresholds and the occurrence of side effects we tested 0-3 additional trajectories with STN-positive MER signals at 2–3 different depths, each. Intraoperative clinical testing graded finger tapping and fast, alternating pronation/supination movements of the hands for bradykinesia as well as standardized assessment of rigidity and tremor according to the United Parkinson’s Disease Rating Scale Part III (UPDRS). Improvements were documented in 25% steps as compared to baseline for every amplitude applied.

Documentation of side effects included dysarthria, contralateral facial or limb contractions, paresthesia, diplopia and ptosis as well as conjugated eye deviations. Improvements of symptoms were expected to start at 1–2 mA. Testing for side effects was stopped as soon as reproducible side effects occurred or extended up to a maximum of 6 mA stimulation intensity. The optimal intraoperative stimulation site was defined as the location with the earliest reduction of symptoms and a high threshold for side effects. Having found the optimal stimulation site, the FHC electrode was removed and replaced by a quadripolar (3389, Medtronic, Minneapolis, MN) or octopolar (Abbott (former St. Jude Medical)), or Boston Scientific (Marlborough, US) DBS electrode under fluoroscopic control.

Intraoperative disorientation was determined by patients’ sudden unawareness of their surroundings, including (i) head fixation in stereotactic frame, (ii) recognition of the treating physicians, (iii) understanding of the presented exercises, and (iv) tendency of trying to get up from the operating table.

### Statistical analysis

Demographic and clinical data was analyzed using one-sided Fisher’s exact tests and *t*-tests. The presence or absence of somnolence and disorientation, as well as application of apomorphine and sedative drugs were encoded binarily. For analyses of frequencies of categorical parameters, two-tailed Chi square tests and Fischer’s Exact tests were performed. Correlation between sedation and intraoperative somnolence was also analyzed by logistic regression analysis. All statistical analysis was performed using IBM SPSS Statistics software (versions 25 and 29, Armonk, NY, IBM Corp). A *p*-value of ≤ 0.05 was considered statistically significant.

## Results

### Sedative drugs

In total, 21 patients received sedative drugs (propofol and remifentanil) during skin incisions and burr hole trepanations. The remaining 101 patients were operated without or only with minimal sedation during surgery. In the sedated group, 38.1% (8/21) of the patients presented with somnolence during clinical testing after electrode placement on the first side. In the non-sedated group, somnolence occurred in 14.9% (15/101) on the first operated side (*p*=0.019, Table 1). On the second operated side, 52.4% (11/21) in the sedated group versus 18.8% (19/101) in the non-sedated group presented with somnolence during clinical testing (*p*=0.004, Table 1). No significant difference could be observed for the occurrence of intraoperative disorientation (*p*=0.284, Table 1).

**Table 1:** Factors influencing the occurrence of intraoperative somnolence/disorientation during awake DBS surgery

		Somnolence 1st operated side		Somnolence 2nd operated side		Disorientation	
		Yes (%)	*p*	Yes (%)	*p*	Yes (%)	*p*
Sedative drugs	Yes (*n*=21)	38.1	***0.019***	52.4	***0.004***	19.1	*0.284*
	No (*n=*101)	14.9		18.8		11.9	
Apomorphine	Yes (*n=*81)	16.0	*0.192*	18.5	***0.026***	9.9	*0.115*
	No (*n=*41)	24.4		36.6		19.5	
MRI on day of surgery*	Yes (*n=*9)	23.2	*0.226*	55.6	***0.040***	22.2	*0.335*
	no (*n=*113)	17.7		22.1		12.4	

Significant *p*-values are highlighted in bold

^*^Under general anesthesia

### Peri-operative apomorphine

In 41 patients, oral L-DOPA medication was withdrawn one day prior to surgery without any subcutaneous apomorphine substitution (no-apomorphine group). Of these 41 patients in the no-apomorphine group, 21 received sedative drugs during surgery. In the remaining 81 patients, subcutaneous apomorphine was started several days prior to surgery and stopped one hour before the procedure (apomorphine group). All patients who received perioperative apomorphine were in the non-sedated group.

After the insertion of microelectrodes on the first operated side, 24.4% (10/41) patients of the no-apomorphine group developed somnolence, whereas 16.0% (13/81) in the apomorphine group presented with somnolence (*p*=0.192, Table 1). This trend was significant for the second operated side, where 36.6% (15/41) of patients in the no-apomorphine group developed somnolence and 18.5% (15/81) of the apomorphine group presented with this symptom (*p*=0.026, Table 1). Again, for intraoperative disorientation, no significant difference could be observed (*p*=0.115, Table 1).

To test for the bias caused by the sedation in the non-apomorphine group, we then analyzed the effect of apomorphine application only for patients in the non-sedated group. No significant difference could be observed (*p*=0.390 and *p*=0.550 for somnolence at first and second operated side, respectively).

### MRI on the day of surgery

Nine patients received the MRI for stereotactic planning under general anesthesia on the day of surgery, while for 113 patients the MRI was performed a few days prior to the procedure (under general anesthesia). While no significant difference of the occurrence of somnolence could be observed after the first operated side between the two groups (*p*=0.226, Table 1), the performance of the MRI on the day of the procedure resulted in significantly higher incidence of somnolence after the second operated side (55.6% versus 22.1%, *p*=0.040, Table 1). No difference could be observed for the occurrence of intraoperative disorientation.

As the use of (i) sedatives, (ii) the application of apomorphine, and (iii) performing the MRI on the day of the surgery significantly correlated with the occurrence of intraoperative somnolence, we further tested patient and procedural characteristics related to these variables. Specifically, we analyzed the age, duration of Parkinson’s disease prior to DBS surgery, and the width of the 3^rd^ ventricle (as a marker for brain atrophy). Concerning the extent of L-DOPA dependency, we included UPDRS scores (off-medication), UPDRS improvement (in %) and the pre-operative L-DOPA equivalent dose in the analysis. As the performance of the MRI on the day of the surgery increased the duration of the procedure, we included the duration of the surgical and the complete procedure, the beginning of the surgery (in minutes after midnight) and the number of MER used.

The occurrence of intraoperative disorientation did not associate with any of the variables mentioned above. Therefore, only the occurrence of intraoperative somnolence was evaluated in the following analyses.

### Age

While no difference in the occurrence of somnolence could be seen after the first operated side in the sedated group, patients presenting with somnolence after placement of microelectrodes on the second operated side were significantly older than patients in whom somnolence did not occur (64.0 ± 6.4 years versus 61.0 ± 7.4 years, *p*=0.039, Table 2). No correlation could be seen in the non-sedated group (*p*>0.05, Table 3).

**Table 2:** Correlation of analyzed variables with occurrence of intraoperative somnolence during DBS surgery in all patients (*n=*122)

	Somnolence 1st operated side			Somnolence 2nd operated side		
	Yes (*n=*23)	No (*n=*99)	*P*	Yes (*n=*30)	No (*n=*92)	*P*
Age (years)	63.6(± 5.7)	61.46(± 7.5)	*0.177*	64.0(± 6.4)	61.0 (± 7.4)	***0.039***
Duration of disease (years)	12.0(± 4.0)	11.2(± 4.6)	*0.402*	12.5(± 4.0)	10.9 (± 4.6)	*0.080*
UPDRS (off-medication)	49.0(± 20.1)	43.8(±15.0)	*0.248*	50.0(± 18.9)	43.0 (± 14.8)	*0.071*
% UPDRS improvement	67.6(± 15.0)	62.3(±16.7)	*0.152*	68.1(± 13.5)	61.9(±17.1)	***0.046***
Width 3rd ventricle (mm)	6.7(± 2.4)	6.9(±2.3)	*0.692*	6.7(± 2.3)	6.9 (±2.3)	*0.713*
L-DOPA equivalent dose (mg)	1177.4(±384.5)	1077.0(±441.2)	*0.663*	1100.0(±389.6)	1079.7 (±441.2)	*0.812*
Beginning of surgery (min after midnight)	654.7(± 47.7)	636.2(± 38.2)	***0.049***	657.0(± 50.8)	634.1(± 35.2)	***0.027***
Duration of surgery (min)	263.1(± 62.8)	249.5(± 50.9)	*0.341*	266.8(± 66.1)	247.3(± 47.9)	*0.082*
Duration of complete procedure (min)	437.2(± 102.4)	406.8(± 78.4)	*0.193*	443.4(± 109.1)	402.5(± 71.6)	***0.020***

Significant *p*-values are highlighted in bold

**Table 3:** Correlation of analyzed variables with occurrence of intraoperative somnolence during DBS surgery in the non-sedated group (*n*=101)

	Somnolence 1st operated side			Somnolence 2nd operated side		
	Yes (*n=*15)	No (*n=*86)	*P*	Yes (*n=*19)	No (*n=*82)	*p*
Age (years)	63.1(± 6.0)	61.4(±7.6)	*0.333*	64.1(± 7.0)	61.1(± 7.4)	*0.112*
Duration of disease (years)	11.0(±4.3)	11.0(±4.6)	*0.985*	12.1(± 4.6)	10.8(± 4.5)	*0.284*
UPDRS (off-medication)	46.5(± 20.8)	41.1(±13.7)	*0.407*	46.4(± 18.9)	40.8(± 13.8)	*0.221*
% UPDRS improvement	68.1(± 16.1)	62.5(±17.4)	*0.237*	69.2(± 14.7)	61.9(± 17.5)	*0.072*
Width 3rd ventricle (mm)	6.4(±2.2)	6.9(±2.3)	*0.481*	6.6(± 2.3)	6.9(±2.2)	*0.712*
L-DOPA equivalent dose (mg)	1142(± 393)	1089(± 453)	*0.648*	1147(± 403)	1085(±454)	*0.636*
Beginning of surgery (min after midnight)	632(± 13)	626(±16)	*0.203*	629(±10)	627 ± 16)	*0.327*
Duration of surgery (min)	234(±51)	237(± 38)	*0.822*	231(± 45)	238(± 39)	*0.484*
Duration of complete procedure (min)	385(± 53)	384(± 40)	*0.967*	379(± 45)	386(±42)	*0.578*

### Duration of disease prior to surgery

There was no significant correlation of the intraoperative somnolence and the duration of PD prior to the DBS surgery. However, a trend could be seen for the second operated side (12.5 ± 4.0 years versus 10.9± 4.6 years, *p* = 0.080, Table 2). No correlation could be seen in the non-sedated group (*p*>0.05, Table 3).

### UPDRS prior to surgery: off-medication-state and extent of improvement

The preoperative UPDRS in off-medication-state did not significantly correlate with the occurrence of intraoperative somnolence on the first operated side (*p*=0.248), while a trend was observable for the second operated side (*p*=0.071, Table 2). For the extent of UPDRS improvement (in %), a significant correlation could be seen for the second operated side (*p*=0.046, Table 2). No correlation was found in the non-sedated group (*p*>0.05, Table 3).

### Width of 3rd ventricle (in [mm])

The width of the third ventricle as an indirect indicator for brain atrophy did not seem to have any influence on the occurrence of somnolence during the procedure (*p*>0.05, Table 2). No correlation could be seen in the non-sedated group (*p*>0.05, Table 3).

### Pre-operative L-DOPA equivalent dose (in [mg])

The amount of the pre-operative L-DOPA equivalent dose did not significantly differ between the patients with or without intraoperative somnolence (*p*>0.05, Table 2). No correlation could be seen in the non-sedated group (*p*>0.05, Table 3).

### Beginning of surgery (in [min] after midnight)

The beginning of the surgical procedure (as the time of first skin incision after midnight) was significantly later in the patients presenting with somnolence after both, the first and the second operated side (*p*=0.049 and *p*=0.027, respectively, Table 2). No correlation could be seen in the non-sedated group (*p*>0.05, Table 3).

### Duration of surgical and complete procedure (in [min])

The duration of the surgical DBS procedure (from first skin incision to last skin closure) did not significantly correlate with the occurrence of intraoperative somnolence (*p*=0.341 and *p*=0.082 for first and second side respectively, Table 2). However, the overall time of the procedure on the day of the surgery (starting with the application of the stereotactic frame and ending with the last skin closure) was significantly longer in patients who developed somnolence on the second operated side (*p*= 0.020, Table 2). No correlation could be seen in the non-sedated group (*p*>0.05, Table 3).

### Number of microelectrode trajectories

**Fig. 1:**
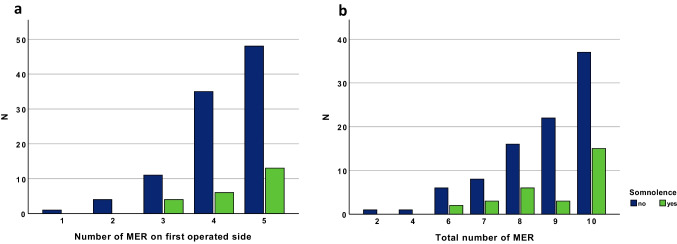
The occurrence of somnolence based on number of MER implanted for testing on **a**) the first operated side and **b**) second operated side (total number of MER)

Intraoperative somnolence did not occur in patients with few MER (1 or 2 MER on the first operated side, 2 or 4 total MER). 21.3%, 14.6%, and 26.7% of patients receiving 5, 4, or 3 MER on the first operated side presented with intraoperative somnolence, respectively (Fig. 1). Taking the number of MER trajectories together, somnolence occurred in 28.8% of patients with 10 MER trajectories and 12.0% of patients with 9 MER. Patients with 5 MER trajectories on the first operated side presented significantly more often with intraoperative somnolence compared to patients with 1–3 MER (*p*=0.041, Fig. 1). When looking at the total number of MER trajectories, patients with 10 trajectories (*n*=52) tended to present more frequently with somnolence (28.8% versus 12.0%) compared to patients with a total of 9 trajectories (*n*=25, *p*=0.102, Fig. 1).

The occurrence of disorientation was found more frequently in patients with 10 trajectories (19.2%) than in patients with 9 (8.0%; *p*=0.023) or in patients with 8 trajectories (9.1%; *p*=0.041).

### AC-PC coordinates of the planned targets

Next, we wanted to test whether electrode placement might have influenced the occurrence of intraoperative somnolence. Regarding the whole collective, the planned targets tended to be more medial (*p*=0.060) and more inferior (*p*=0.051, Suppl. Table 1) in patients with somnolence and disorientation as in patients who did not deteriorate. In the anterior-posterior direction (y-coordinate) no differences were found (Suppl. Table 1). We then analyzed the sedated and non-sedated subgroups separately and found no significant differences concerning the x-, y-, z-coordinates in the non-sedated group. In the sedated group the planned target coordinates were significantly more medial (*p*=0.045) and more inferior (*p*=0.052) in patients with intraoperative somnolence (Suppl. Table 2).

To account for the use of sedative drugs, we then analyzed the patient and procedural characteristics for the non-sedated subgroup only. No significant correlation could be observed, indicating that the use of sedative drugs is the main contributing variable for the occurrence of intraoperative somnolence during DBS for PD patients. Logistic regression analysis confirmed a significant correlation between the use of sedative drugs and intraoperative somnolence (*p*=0.017 and *p*=0.002 for first and second operated side, respectively) with an odds ratio of 3.528 (95% confidence interval 1.250–9.957) and 4.747 (95% confidence interval 1.762–12.791) for first and second operated side, respectively. Patients that presented with intraoperative somnolence were therefore 3.528 to 4.747 times more likely to have received sedative drugs during the DBS procedure.

## Discussion

In awake DBS procedures, somnolence and disorientation may occur during the operation which might hamper the reliability of clinical testing, performed to find the optimal stimulation area for the individual patient [4, 11, 15, 25, 31].

In deep brain stimulation, the validity and necessity of intraoperative clinical testing has been questioned repeatedly [2]. The majority of comparative studies concerning awake versus asleep procedures did not find significant differences with regard to the postoperative benefit of the patients [6, 8, 9, 13, 14, 18–20, 22, 27, 33, 37]. However, the execution of awake DBS procedures is quite heterogeneous, if at all reported in detail, which makes a generalization of the results difficult.

In this study, we show that the usage of analgesics and sedatives during DBS surgery is the main influencing factor for the occurrence of intraoperative somnolence which reduces the reliability and validity of intraoperative clinical testing. In our institution, we have developed an interdisciplinary approach to awake DBS surgery with psychological support for the patients by the anesthesiologist [24, 40]. We initially tended to assume that we increase the patients’ comfort by the application of intra-operative analgesics and sedatives to avoid the stress during skin incisions and burr hole trepanation. Yet, we observe a much greater discomfort when patients wake up after sedation in an unfamiliar environment. Pulse rate and blood pressure then rise significantly and are combined with a palpable state of anxiety, which further hampers the reliability of intra-operative testing [16]. Omitting the drugs and providing psychological assistance during the whole procedure significantly reduced autonomic and mental stress reactions. Clinical testing can start immediately when needed and without the restriction of reduced vigilance and compliance due to prior sedation [24, 40].

While the use of sedatives seems to be the main contributing factor, prolonged L-DOPA deprivation and any means increasing the time of the DBS procedure might also influence the occurrence of intraoperative somnolence, thus hindering meaningful intraoperative clinical testing. In this study we found that the drastic reduction of the period of dopamine deprivation by using perioperative subcutaneous apomorphine application reduced the occurrence of somnolence during the procedure. Similarly, the later the surgery starts and the longer the operation lasts, the longer the time of dopamine deprivation will be. In addition, patients are prone to get tired in long procedures which further reduces compliance and vigilance. It is not surprising that patients who went through MRI scans under general anesthesia on the day of surgery, following a night without dopamine substitution and having received sedative drugs during the operation were those with the highest rates of somnolence and disorientation.

We assumed that the number of microelectrode trajectories in a patient would correlate with the incidence of somnolence and disorientation as the length of the clinical testing, MER recording, and therefore the procedure increases with a greater number of trajectories. Also, more severe trauma to the brain is caused [17, 41]. Yet, the correlation was not as clear as anticipated. In the total collective, we found a significantly higher incidence of somnolence and disorientation in patients with 5 compared to patients with 1-3 trajectories on the first operated side. This might be explained by the low number of patients with only 1–3 MER trajectories, as the vast majority of our patients had received 4 or 5 trajectories on the first operated side and 8, 9, or 10 trajectories taken both sides together.

There are several limitations in this study, mainly that some of the subgroups were mutually exclusive (e.g., sedation and apomorphine treatment) which hinders the statistical comparison of these groups. Another influencing factor, which was not investigated in the present study was the path of the trajectories of the electrodes through the brain. For example, Witt et al. pointed out that if the electrodes traverse the caudate nucleus postoperative cognitive deficits might occur [38], even though the study results could not be replicated by Bot et al. [7].

The goal of awake DBS surgery includes not only the functional verification of the ideal electrode placement as determined by MRI and anatomy, but to clinically determine optimal electrode placement with a good clinical benefit (minimal stimulation needed with wide therapeutic range) and minimization of adverse effects before permanent implantation. This benefit of awake DBS procedures requires a vigilant and cooperative patient for intraoperative testing. The present study sheds light on possible influencing factors (i.e., use of sedatives, increased length of DBS procedure, prolonged L-DOPA withdrawal) that might be accountable for the occurrence of intraoperative somnolence and disorientation, both of which limit the reliability of clinical testing in awake DBS surgery.

As these factors influence patients’ intraoperative vigilance and cooperation, they need to be considered individually when planning patients’ DBS procedures. If they cannot be omitted or considered due to institutional or personal/patient requirements or circumstances, the advantages of awake surgery might be lost and an asleep procedure might be non-inferior to the awake surgery with intraoperative testing.

However, when awake DBS surgery is performed, especially the use of sedatives should be avoided by any means.

## Supplementary information

ESM 1 (DOCX 16 kb)

ESM 2 (DOCX 16 kb)

## Abbreviations

AC-PC line: Anterior commissure - posterior commissure line
DBS: Deep brain stimulation
MER: Microelectrode recording
PD: Parkinson’s disease
STN: Subthalamic nucleus
UPDRS: Unified Parkinson’s Disease Rating Scale
