# Supplementary material for: Factors influencing the reliability of intraoperative testing in deep brain stimulation for Parkinson’s disease
Source: Acta Neurochir (Wien). 2023 Jun 2;165(8):2179–87. doi: 10.1007/s00701-023-05624-4 (PMC10409887; doi:10.1007/s00701-023-05624-4)
Supplement: Supplementary file 1 — (DOCX 16 kb) [file 701_2023_5624_MOESM1_ESM.docx]

**Suppl. Table 1**

**Mid AC-PC coordinates of planned targets for patients with and without intraoperative somnolence/disorientation (n=122)**

|  | **Somnolence after 1st operated side** | | | **Somnolence after 2nd operated side** | | | **Disorientation** | | |
| --- | --- | --- | --- | --- | --- | --- | --- | --- | --- |
|  | yes (n=23) | no (n=99) | *p* | yes (n=30) | no (n=92) | *p* | yes (n=16) | no (n=106) | *p* |
| **xTarget, left** | -11.8  (+/-1.4) | -12.3  (+/-1.2) | *0.861* | -11.8  (+/- 1.3) | -12.3  (+/-1.2) | *0.060* | -11.9  (+/-1.3) | -12.2  (+/-1.2) | *0.365* |
| **xTarget,right** |  |  |  | 12.3  (+/- 1.0) | 12.5  (+/-1.0) | *0.506* | 12.2  (+/-1.1) | 12.5  (+/-1.0) | *0.364* |
| **yTarget, left** | -1.7  (+/-1.1) | -1.6  (+/- 0.8) | *0.752* | -1.8  (+/-1.0) | -1.6  (+/-0.8) | *0.175* | -1.5  (+/-0.9) | -1.6  (+/- 0.8) | *0.584* |
| **yTarget, right** |  |  |  | -1.5  (+/-10.9) | -1.4  (+/-0.8) | *0.648* | -1.2  (+/-0.9) | -1.5  (+/0.8) | *0.238* |
| **zTarget, left** | -5.3  (+/-0.9) | -5.1  (+/-0.8) | *0.399* | -5.4  (+/-0.9) | -5.1  (+/-0.8) | *0.152* | -5.5  (+/-0.7) | -5.1  (+/-0.9) | *0.051* |
| **zTarget, right** |  |  |  | -5.0  (+/-0.8) | 5.1  (+/-0.8) | *0.637* | -5.2  (+/-0.7) | -5.0  (+/-0.8) | *0.391* |
