# Supplementary material for: Factors influencing the reliability of intraoperative testing in deep brain stimulation for Parkinson’s disease
Source: Acta Neurochir (Wien). 2023 Jun 2;165(8):2179–87. doi: 10.1007/s00701-023-05624-4 (PMC10409887; doi:10.1007/s00701-023-05624-4)
Supplement: Supplementary file 2 — (DOCX 16 kb) [file 701_2023_5624_MOESM2_ESM.docx]

**Suppl. Table 2**

**Mid AC-PC coordinates of planned targets for patients with and without intraoperative somnolence/disorientation in the sedated group (n=21)**

|  | **Somnolence 1st operated side** | | | **Somnolence 2nd operated side** | | | **Disorientation** | | |
| --- | --- | --- | --- | --- | --- | --- | --- | --- | --- |
|  | yes (n=8) | no (n=13) | *p* | yes (n=11) | no (n=10) | *p* | yes (n=17) | no (n=4) | *p* |
| **xTarget, left** | -11.1  (+/- 1.9) | -12.6  (+/-1.0) | *0.077* | -11.4  (+/- 1.7) | -12.7  (+/-1.1) | ***0.045*** | -10.6  (+/-1.2) | -12.4  (+/-1.4) | ***0.034*** |
| **xTarget, right** |  |  |  | 12.5  (+/- 1.2) | 12.6  (+/-0.8) | *0.814* | 11.7  (+/-1.5) | 12.7  (+/-0.8) | *0.062* |
| **yTarget, left** | -2.5  (+/-0.7) | -2.6  (+/-1.1) | *0.899* | -2.6  (+/-1.0) | -2.5  (+/-1.1) | *0.808* | -2.5  (+/-0.7) | -2.6  (+/-1.1) | *0.848* |
| **yTarget, right** |  |  |  | -2.0  (+/-0.9) | -2.3  (+/-1.0) | *0.497* | -1.5  (+/-1.1) | -2.2  (+/-0.9) | *0.162* |
| **zTarget, left** | -6.0  (+/-1.1) | -5.0  (+/-1.1) | *0.052* | -5.7  (+/-1.1) | -5.0  (+/-1.2) | *0.218* | -5.8  (+/-0.9) | -5.3  (+/-1.2) | *0.370* |
| **zTarget, right** |  |  |  | -5.0  (+/-1.0) | -5.1  (+/-1.1) | *0.884* | -5.5  (+/-0.8) | -4.9  (+/-1.1) | *0.391* |
